# CK2 Pro-Survival Role in Prostate Cancer Is Mediated via Maintenance and Promotion of Androgen Receptor and NFκB p65 Expression

**DOI:** 10.3390/ph12020089

**Published:** 2019-06-14

**Authors:** Janeen H. Trembley, Betsy T. Kren, Md. J. Abedin, Daniel P. Shaughnessy, Yingming Li, Scott M. Dehm, Khalil Ahmed

**Affiliations:** 1 Research Service, Minneapolis VA Health Care System, Minneapolis, MN 55417, USA; krenx001@umn.edu (B.T.K.); abedin@umn.edu (M.J.A.); shaug028@umn.edu (D.P.S.); 2 Department of Laboratory Medicine and Pathology, University of Minnesota, Minneapolis, MN 55455, USA; dehm@umn.edu; 3 Masonic Cancer Center, University of Minnesota, Minneapolis, MN 55455, USA; lixxx354@umn.edu; 4 Department of Urology, University of Minnesota, Minneapolis, MN 55455, USA

**Keywords:** prostate, CK2, AR, Tenfibgen, mitochondria, survival, cell death, xenograft, CRPC, in vivo delivery, nanoparticle, nanocapsule

## Abstract

The prosurvival protein kinase CK2, androgen receptor (AR), and nuclear factor kappa B (NFκB) interact in the function of prostate cells, and there is evidence of crosstalk between these signals in the pathobiology of prostate cancer (PCa). As CK2 is elevated in PCa, and AR and NFκB are involved in the development and progression of prostate cancer, we investigated their interaction in benign and malignant prostate cells in the presence of altered CK2 expression. Our results show that elevation of CK2 levels caused increased levels of AR and NFκB p65 in prostate cells of different phenotypes. Analysis of TCGA PCa data indicated that AR and CK2α RNA expression are strongly correlated. Small molecule inhibition or molecular down-regulation of CK2 caused reduction in AR mRNA expression and protein levels in PCa cells and in orthotopic xenograft tumors by various pathways. Among these, regulation of AR protein stability plays a unifying role in CK2 maintenance of AR protein levels. Our results show induction of various endoplasmic reticulum stress signals after CK2 inhibition, which may play a role in the PCa cell death response. Of note, CK2 inhibition caused loss of cell viability in both parental and enzalutamide-resistant castrate-resistant PCa cells. The present work elucidates the specific link of CK2 to the pathogenesis of PCa in association with AR and NFκB expression; further, the observation that inhibition of CK2 can exert a growth inhibitory effect on therapy-resistant PCa cells emphasizes the potential utility of CK2 inhibition in patients who are on enzalutamide treatment for advanced cancer.

## 1. Introduction

The continued availability of androgens functioning through their cognate receptor (androgen receptor, AR) is essential for the development and growth of the prostate throughout the lifetime of the individual; likewise, the initial development of prostate cancer (PCa) also involves androgen-dependent AR function [1,2]. In our early investigations into CK2 function, we demonstrated a relationship between androgen function and protein kinase CK2 in prostate [3,4,5,6,7], and subsequently reported that CK2 was elevated in prostate neoplasia [8,9]. Similar observations pertaining to increased CK2 have been made in various cancers studied (see, e.g., [10,11,12]). 

Protein kinase CK2 (formerly casein kinase 2 or II) is a highly conserved and ubiquitous protein serine/threonine kinase broadly localized in both the cytoplasmic and nuclear compartments. It engages in many functions, including roles in normal and disease-associated cell growth and proliferation. Its heterotetrameric structure includes two catalytic subunits (42 kDa α and 38 kDa α′) linked by two regulatory subunits (28 kDa β). CK2 appears to be constitutively active; however, one regulatory mechanism of CK2 signaling relates to its dynamic shuttling in response to altered growth signals [7]. CK2 mediates phosphorylation of numerous substrates involved in gene expression, cell growth, and other cell processes [8,13]. A particularly important feature of CK2 biology is that it is essential for cell survival and attempts to produce CK2α- and CK2β-knockout mice have failed [14,15]. Numerous studies have culminated in the proposal that CK2 is among the master regulators of diverse functions in the cell under normal as well as disease states. CK2 is elevated during normal cell proliferation; however, it demonstrates an increased persistent expression at the protein level in cancer cells [8,10,11,12]. Besides its role in cell growth and proliferation, CK2 also functions to suppress cell death—this aspect may reflect one of the most significant functions of CK2, especially with respect to its role in cancer biology [16,17,18,19]. 

Our early work hinted at a direct crosstalk between AR function and CK2 [20]. A number of recent studies have supported this notion by demonstrating that chemical inhibition of CK2 causes a reduction in AR dependent transcriptional activity as well as loss of AR protein [21,22,23,24]. Inhibition of CK2 or its molecular downregulation results in cancer cell death, including AR-positive and -negative PCa cells. Accordingly, CK2 has been proposed as an important target for PCa therapy [25,26,27,28,29]. Currently, blockade of AR function is the focus of first and second-line therapies for PCa, promoting regression of the tumor (see, e.g., [30,31,32,33,34]). However, it is well known that androgen-insensitive disease re-emerges, referred to as castrate-resistant PCa (CRPC). Given CK2 control over AR levels and the importance of both molecules relating to prostate cell viability, better understanding of the nature of CK2 and AR interactions in PCa cells of different phenotypes is important for progression in the PCa field. This knowledge may further contribute to PCa therapy through strategies combining targeting of CK2 and AR functions. 

NFκB p65 is among the well-established substrates of CK2; CK2 phosphorylates p65 at S529, and this event is associated with NFκB p65 nuclear translocation [35]. Abnormal activation of NFκB p65 has been reported in many cancers [36,37,38,39,40]. In previous work, we observed that nuclear CK2α and NFκB p65 levels are significantly higher in PCa compared with benign prostatic hyperplasia (BPH) and these proteins are positively correlated with each other in both diseases [41]. Further, we have observed that both total NFκB p65 and p65 phosphorylation (especially on S529) can serve as a surrogate for measuring altered CK2 activity in xenograft models of cancer [42,43,44,45].

In the present work, we demonstrate inter-connection of CK2 expression with NFκB p65 and AR in non-transformed prostate cells, unifying aspects of our previous work. We further examined AR and NFκB p65 dynamics in response to altered CK2 expression and activity in various PCa cell lines representing AR-expressing androgen-sensitive and CRPC phenotypes. Our results suggest that modulation of CK2 by molecular and chemical means has distinct effects on AR protein and mRNA expression. CK2 signaling promotes prostate and PCa cell survival via regulation of NFκB p65 and AR protein levels whereas anti-CK2 strategies can cause PCa cell death via multiple mechanisms regardless of the AR-driven nature of the disease. Importantly, CRPC cells that are resistant to enzalutamide (a current therapy for advanced CRPC) exhibit a cell death response to anti-CK2 treatment. Together, these observations have implications for PCa therapy, suggesting a significantly improved response by targeting both AR and CK2. 

## 2. Results

### 2.1. Effect of Increased CK2 Level on AR and NFκB p65 Expression in Benign and Malignant Prostate Cells

**Figure 1:**
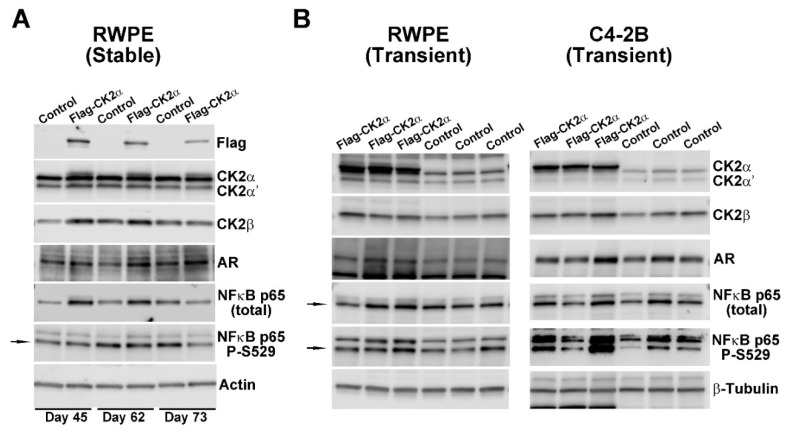
Effects of increased CK2α expression on AR and NFκB p65 protein levels. (**A**) Immunoblot analysis of RWPE-1 cells stably expressing Flag-CK2α after 45, 62 and 73 days in culture. Control lanes represent either parental RWPE-1 cells or RWPE-1 cells stably expressing empty vector collected simultaneously with Flag-CK2α cells. Proteins detected are indicated on the right side of the blots. Actin signal was used as the loading control. (**B**) Immunoblot analysis of RWPE-1 and C4-2B cell lysates 24 h after transient transfection with Flag-CK2α expression construct. Control lanes represent empty vector transfected cells. β-Tubulin signal was used as the loading control. Arrows indicate correct band.

As CK2 protein levels are elevated in PCa, we wanted to determine the effect of CK2 overexpression in non-malignant and malignant prostate cells with respect to the expression of AR and NFκB p65. First, a non-transformed prostate cell line stably expressing FLAG-CK2α using RWPE-1 cells was generated. Immunoblot analysis (Figure 1A) shows the presence of FLAG-CK2α in cells that were in culture from 45 to 73 days. These cells additionally show increased expression of CK2α’, CK2β, AR, and NFκB p65 proteins compared with control cells over the same time period. The mean protein expression levels for “RWPE (stable)” cells are summarized in Table 1, and demonstrate that an approximately 1.5-fold increase in CK2 levels similarly raised AR and NFκB p65 total protein levels. 

These observations were confirmed by transient transfection of the same FLAG-CK2α expression construct into RWPE-1 as well as two PCa cell lines, C4-2B and LNCaP. Transient FLAG-CK2α expression increased 2.9 to 4.4-fold, with higher CK2α’ and CK2β levels again observed over control cells (Figure 1B and Table 1). In all FLAG-CK2α expressing cells, elevated AR protein expression was again noted. In contrast to both stable and transient expression of FLAG-CK2α in non-transformed RWPE-1 cells where NFκB p65 effects were predominantly at the total protein level, transient elevation of FLAG-CK2α in malignant prostate cell lines increased phosphorylation of NFκB p65 at the CK2-specific site, S529, without changing total NFκB p65 levels (Table 1). We also examined Cyclin D1 protein, and found that levels were generally unchanged or decreased.

**Figure 2:**
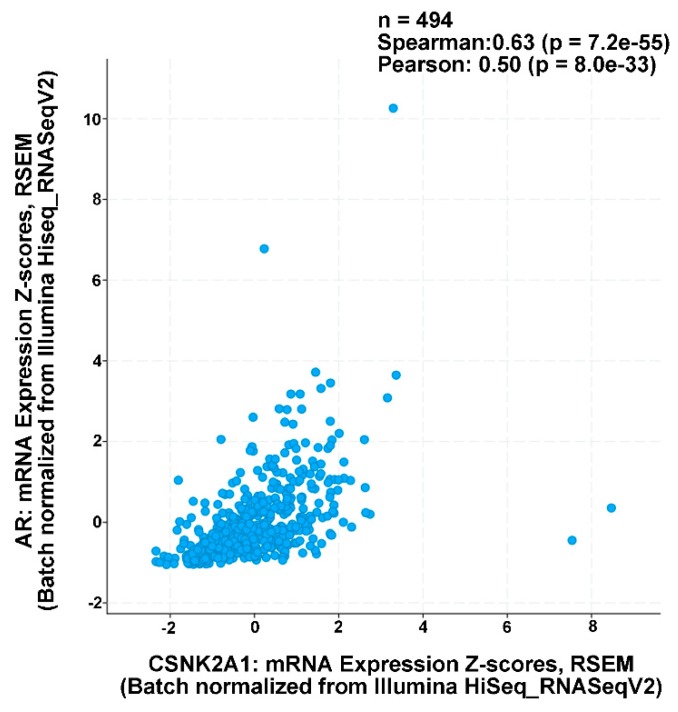
Co-expression of *CSNK2A1* and *AR* genes in prostate cancer patient primary tumor samples. Analysis of co-expression of *CSNK2A1* and *AR* mRNA levels in PCa patient samples from The Cancer Genome Atlas (Pan-Cancer Atlas; n = 494 samples). Correlation analysis and p-values provided within panel. Analysis performed using cBioPortal with Z-score cut-off set at 1.5. RSEM, RNA-Seq by Expectation–Maximization.

To further investigate the relevance of the above-described observations, we decided to determine if a correlation between AR and CK2α RNA expression existed in the tumors of PCa patients. To that end, an analysis of co-expression of *CSNK2A1* and *AR* mRNA levels in PCa patient samples from The Cancer Genome Atlas (TCGA) was performed (Pan-Cancer Atlas; n = 494 samples). The analysis was performed using cBioPortal with a Z-score cut-off set at 1.5, and the result demonstrates a significant positive correlation of the two genes in PCa patient samples (Figure 2) [46,47]. 

### 2.2. Effect of Reducing CK2 Level or Activity on AR Protein Levels in Prostate Cancer Cells 

**Figure 3:**
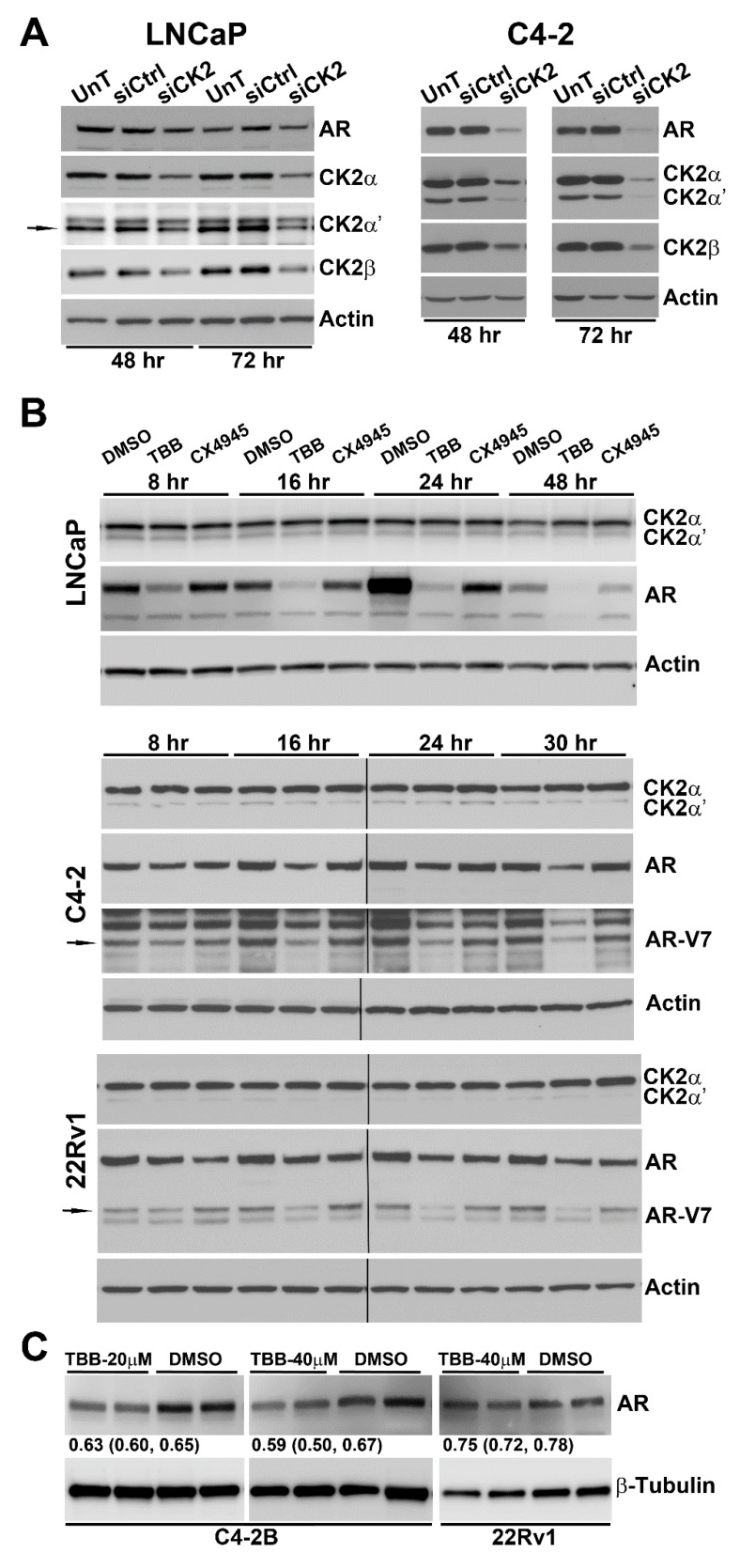
Blocking CK2 expression and activity reduces AR protein levels in PCa cells. (**A**) LNCaP (left panels) and C4-2 (right panels) cells were transfected with CK2αα’-targeted and control siRNAs. Cells were collected 48 and 72 h post-transfection for analysis by immunoblot. (**B**) LNCaP, C4-2, and 22Rv1 cells were treated with 80 µM TBB, 10 µM CX-4945, or equivalent concentration of DMSO. Cells were collects at various time points, as labeled above the lanes, for analysis by immunoblot. UnT = untreated cells. Vertical lines indicate non-contiguous lanes. (**C**) C4-2B and 22Rv1 cells were treated with 20 µM TBB, 40 µM TBB, or equivalent concentration of DMSO. Cells were collected at 24 h for analysis by immunoblot. Mean and 95% confidence intervals for AR protein levels after TBB treatment relative to DMSO treatment are indicated below the AR bands. For all panels: Proteins detected are indicated on the right side of blots, time points analyzed are indicated below the blots, and either actin or β-tubulin were used as loading controls. Arrows indicate correct band.

We determined the effect of reducing the level or activity of CK2 on the abundance of AR in various PCa cells. The results in Figure 3A show the effects of transfecting LNCaP and C4-2 PCa cells with siRNA targeting the CK2α and α’ catalytic subunits. It is apparent that by 72 h post-transfection there is notable loss of AR protein in both cancer cells, although it is more prominent in C4-2 than in LNCaP cells. In Table 2, we present the quantitation of the representative data shown in Figure 3A. 

To test the effect of inhibiting CK2 activity, we employed 80 µM 4,5,6,7-tetrabromobenzotriazole (TBB) or 10 µM CX-4945 in cell culture for varying periods of time. Figure 3B shows the effect of TBB and CX-4945 on LNCaP, C4-2 and 22Rv1 cells treated for 8, 16, 24, and 30 or 48 h. Both inhibitors of CK2 reduced the level of AR protein although the effect of TBB was more pronounced than that of CX-4945. The strong effect of CK2 inhibition by TBB on AR protein abundance was also evident at 24 h using lower concentrations of TBB in C4-2B and 22Rv1 cells (Figure 3C). We also detected reduction in the AR-V7 isoform in both C4-2 and 22Rv1 cells, using a specific AR-V7 antibody (Figure 3B). The androgen-responsive LNCaP cells were more responsive to CK2 inhibition, especially by TBB, than the androgen-insensitive C4-2 and 22Rv1 cells. In Table 3, we present the quantitation of the data shown in Figure 3B. 

### 2.3. Effect of TBB or siRNA-mediated CK2 Inhibition on AR mRNA Levels in Prostate Cancer Cells

To determine whether reducing CK2 activity or expression level had any effect on the AR transcript, we measured AR mRNA levels following treatment of 22Rv1, C4-2, and LNCaP cells with CK2 inhibitor or siCK2 targeting CK2αα΄ (Table 4). The results demonstrate that small molecule inhibition of CK2 activity caused reduction of AR mRNA over time. On the other hand, the treatment of C4-2 and LNCaP cells with siCK2 reduced AR mRNA only slightly in LNCaP cells, and increased AR mRNA levels in C4-2 cells. 

### 2.4. Effect of Reducing CK2 Level or Activity on NFκB p65 Protein Levels and Activation in Prostate Cancer Cells

**Figure 4:**
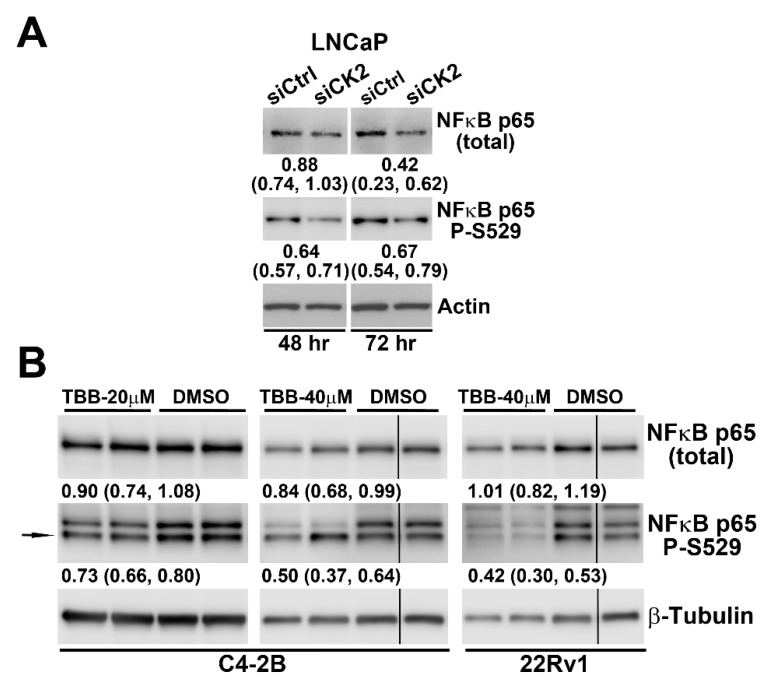
Blocking CK2 expression and activity reduces NFκB p65 protein levels and activation in PCa cells. (**A**) LNCaP cells were transfected with CK2αα’-targeted and control siRNAs. Cells were collected 48 and 72 h post-transfection, as labeled below the blots, for analysis by immunoblot. (**B**) C4-2B and 22Rv1 cells were treated with 20 µM TBB, 40 µM TBB or equivalent concentration of DMSO as indicated. Cells were collected at 24 h for analysis by immunoblot. Vertical lines indicate non-contiguous lanes. For all panels: proteins detected are indicated on the right side of blots and either actin or β-tubulin were used as loading controls. Arrows indicate correct band. Means and 95% confidence intervals for values relative to siControl or DMSO are indicated below each blot.

We examined the impact on NFκB p65 protein levels and activation level in LNCaP cells after transfecting with siRNA targeting the CK2α and α´ catalytic subunits. Reduced activation of NFκB p65 at the CK2 phosphorylation site S529 is evident and equivalent at 48 and 72 h; whereas, loss of total NFκB p65 protein is not notable until 72 h post-transfection (Figure 4A). We also determined the effects of TBB treatment for 24 h on NFκB p65 total protein and activation status at S529. In C4-2B cells, there was a dose and time-dependent slight loss of NFκB p65 total protein, as well as a greater loss of p65 phosphorylation at S529. In 22Rv1 cells, the impact of CK2 inhibition was only observed at the NFκB p65 S529 phosphorylation level at 24 h.

### 2.5. Effect of Reducing CK2 Level in vivo on AR and NFκB p65 Protein Levels in Xenograft Prostate Cancer Tumors

**Figure 5:**
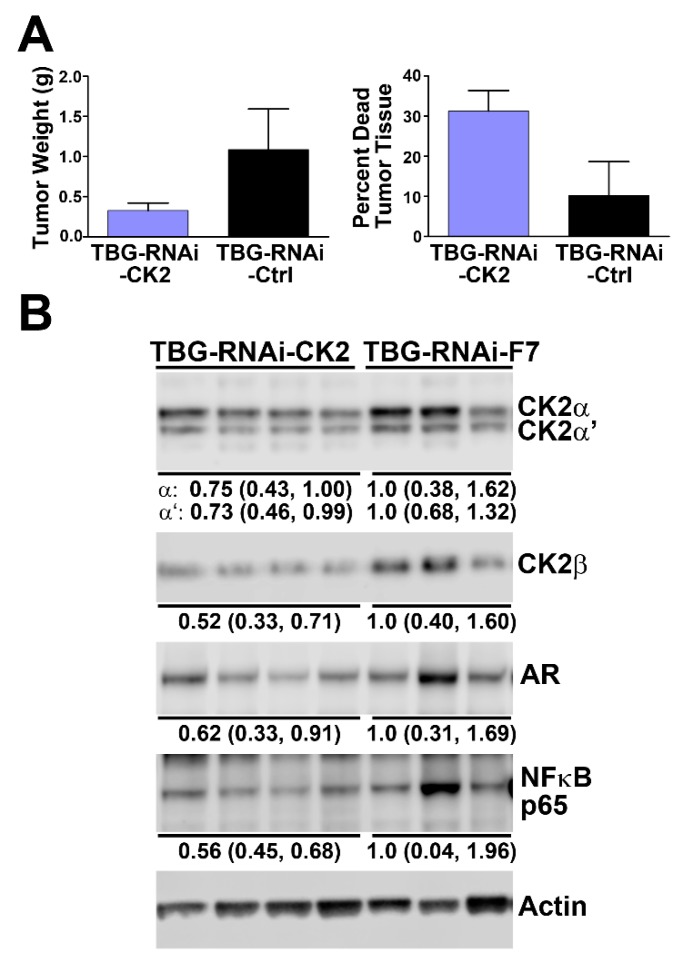
Anti-CK2 nanocapsule RNAi-based systemic treatment reduces expression of CK2, AR and NFκB p65 in 22Rv1 orthotopic xenograft tumors. (**A**) Orthotopic 22Rv1 tumors were initiated in NOD SCID gamma castrated male mice. When tumors were palpable, mice were treated on days 1, 4 and 7 with TBG-RNAi-CK2 or TBG-RNAi-F7 (control) nanocapsule by tail vein injection (0.02 mg/kg). Tumors were harvested on day 8, 24 h after the last treatment. Tumors were weighed, dissected to remove dead tissue, and reweighed. Mean tumor weights per group are indicated on the left panel and the mean percent of dead tumor tissue removed is indicated on the right panel. TBG-RNAi-CK2, n = 4; TBG-RNAi-F7, n = 3. Error bars indicate standard error. (**B**) Orthotopic xenograft 22Rv1 tumors from mice treated with TBG-RNAi-CK2 or TBG-RNAi-F7 (control) nanocapsule drugs were subjected to immunoblot analysis. Treatments are indicated above the lanes, and proteins detected are indicated to the right of the panels. Mean expression levels and 95% confidence intervals are indicated below the individual blots. Actin was used as a loading control.

To establish that the results in cell culture models were also observed in vivo for PCa, we employed orthotopic xenograft 22Rv1 tumors generated in NOD SCID gamma castrated male mice. Treatment of the mice with RNAi-CK2 delivered via a malignant cell-targeted nanocapsule (TBG-RNA-CK2 [44]) resulted in decreased tumor weight and increased dead tumor tissue (Figure 5A). Corresponding to this result is the observed concomitant loss of CK2 subunits as well as reduced AR and NFκB p65 total protein levels (Figure 5B). AR-V7 levels were reduced by TBG-RNAi-CK2 treatment to a lesser extent than full length AR in these tumors (0.92; data not shown). These results corroborate those observed in cultured cells, and thus further support the notion of an interaction of these signals in vivo.

### 2.6. Androgen is Not Required for Effects on AR Status by CK2 Modulation 

**Figure 6:**
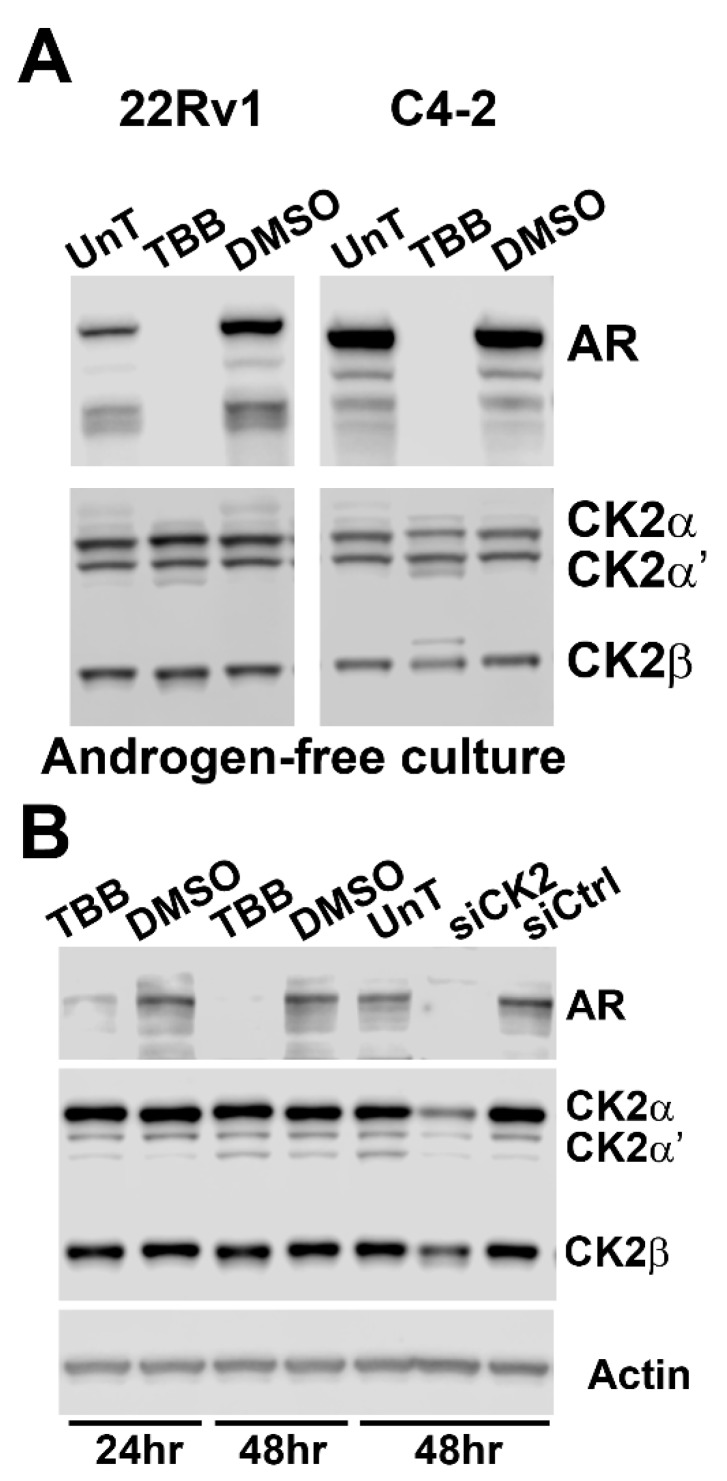
Androgen is not required for CK2 block-mediated loss of AR protein levels, and AR loss is equivalent in non-malignant RWPE-1 cells. (**A**) C4-2 and 22Rv1 cells grown under androgen-free conditions were treated with 80 µM TBB or equivalent concentration of DMSO. Cells were collected 48 h post-treatment for analysis by immunoblot. (**B**) RWPE-1 cells were treated with 80 µM TBB or equivalent concentration of DMSO or transfected with CK2αα’-targeted and control siRNAs. Cells were analyzed by immunoblot at the time points indicated below the panels. For all panels: treatment conditions are indicated above the lanes, and proteins detected indicated to the right of the panels, and actin was used as a loading control.

To test if the presence of androgen had any effect on response of AR to CK2 modulation in prostate tumor cells, we examined the effect of 80 µM TBB on C4-2B and 22Rv1 cells cultured in androgen-free media for a total of 72 h and treated with TBB for 48 h. The results in Figure 6A indicate that the absence of androgen did not influence the reduction of AR protein in response to inhibition of CK2 activity. 

### 2.7. AR Loss is Equivalent in Non-malignant RWPE-1 Cells upon CK2 Inhibition or Loss

We also examined the effects of siRNA- and TBB-mediated reduction in CK2 levels or activity in non-malignant RWPE-1 prostate cells on AR protein expression. The results shown in Figure 6B demonstrate dramatic loss of AR protein after CK2 expression or activity loss. 

### 2.8. Effect of Altered CK2 Expression and Activity on AR Protein Half-life and ER Stress

**Figure 7:**
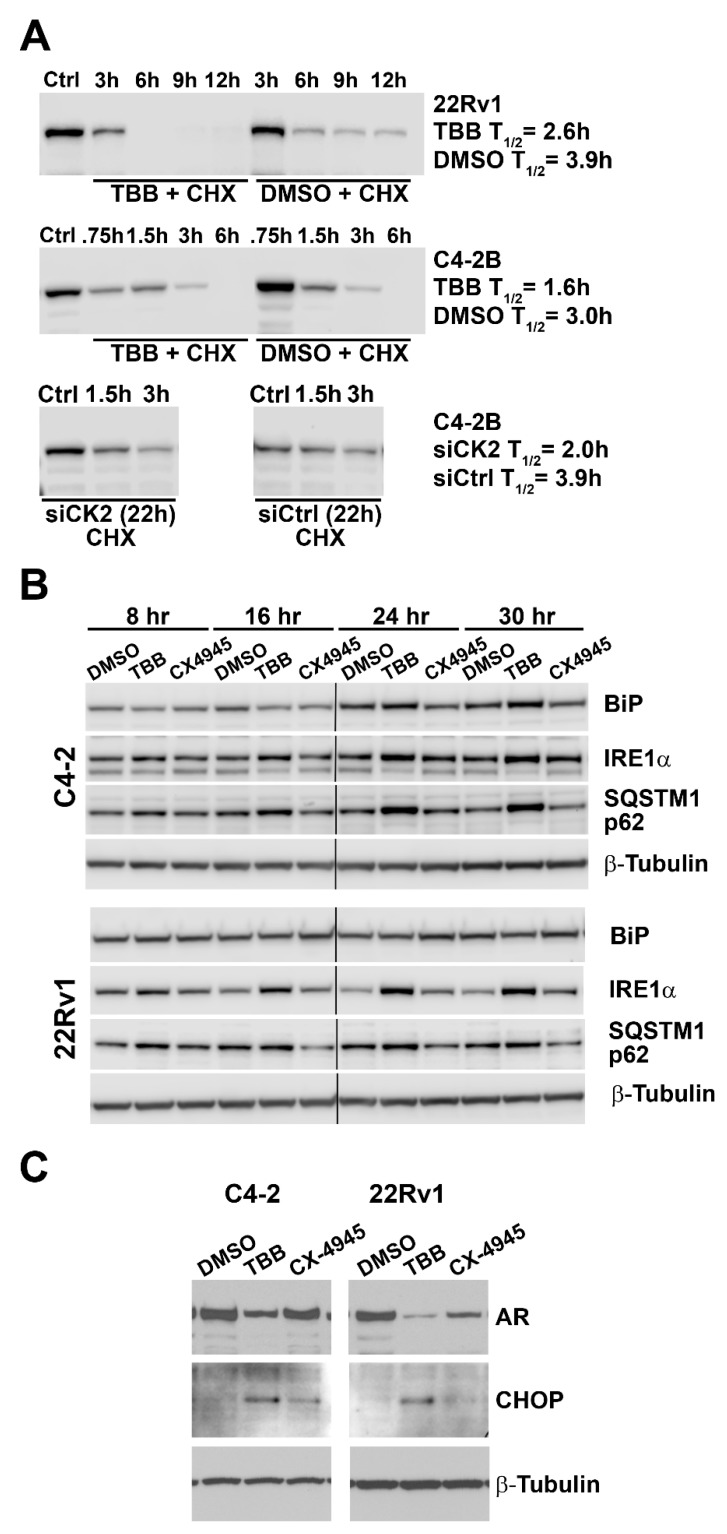
CK2 expression and activity influence AR protein half-life and ER stress signaling. (**A**) 22Rv1 and C4-2B cells were treated with 80 µM TBB or equivalent concentration of DMSO. C4-2B cells were also transfected with CK2αα′-targeted and control siRNAs. Twenty-four hours after TBB or DMSO treatment or 22 h post-transfection, cycloheximide was added to the culture plates, and cells were collected over time as indicated above the blot lanes. Cell lysates were analyzed by immunoblot for AR protein expression, and AR half-life was calculated by linear regression of the quantitated signals. (**B**) C4-2 and 22Rv1 sample lysates from the experiments presented in Figure 3B were analyzed for markers of ER stress and autophagy by immunoblot. **(C)** AR and CHOP signals were analyzed by immunoblot in C4-2 and 22Rv1 48 h lysates following treatment with TBB or CX-4945.

To investigate possible mechanisms of the effects of altered CK2 status on AR abundance, we determined the half-life of AR protein. PCa cells were treated with 80 µM TBB and cycloheximide simultaneously, and cells were collected at various time points as indicated in Figure 7A (upper two panels). C4-2B cells were also transfected with siCK2, and 22 h post-transfection cycloheximide was added and cells were collected at various time points as indicated in Figure 7A (lowest panels). The results in Figure 7A demonstrate that AR half-life is reduced by 34–49% in PCa cells treated with CK2 inhibitor or siCK2. 

Reducing CK2 expression and activity is known to induce changes in the endoplasmic reticulum (ER) stress pathway in cancer cells [48,49,50]. We examined the response of various ER stress signals in C4-2 and 22Rv1 cells following treatment with CK2 inhibitors TBB and CX-4945 over time. Induction of BiP protein was observed at less than 2-fold increases; with TBB-mediated induction in C4-2 cells at 24 and 30 h (Figure 7B), and both TBB- and CX-4945-induced BiP levels in 22Rv1 cells at 20 h (data not shown). IRE1α levels increased 1.2- to 3.3-fold in both PCa cell lines in response to treatment with either CK2 inhibitor and at all time points examined (Figure 7B). Elevated SQSTM1 p62 levels (1.3- to 2.4-fold) were observed in both PCa cell lines, but only with TBB. A somewhat lesser induction of SQSTM1 p62 was observed, 1.1-fold in C4-2B and 2.4-fold in 22Rv1, with 24 h TBB treatment at 40 μM (data not shown). While CHOP induction was detected on immunoblots as early as 8 h post-treatment, we have shown 48 h data due to high background at earlier time points (Figure 7C). CHOP induction at 48 h ranged from 2.4- to 10.8-fold increase, with TBB inducing the biggest changes. 

### 2.9. Effect of Inhibition of CK2 on Viability of Enzalutamide-resistant C4-2B Cells

**Figure 8:**
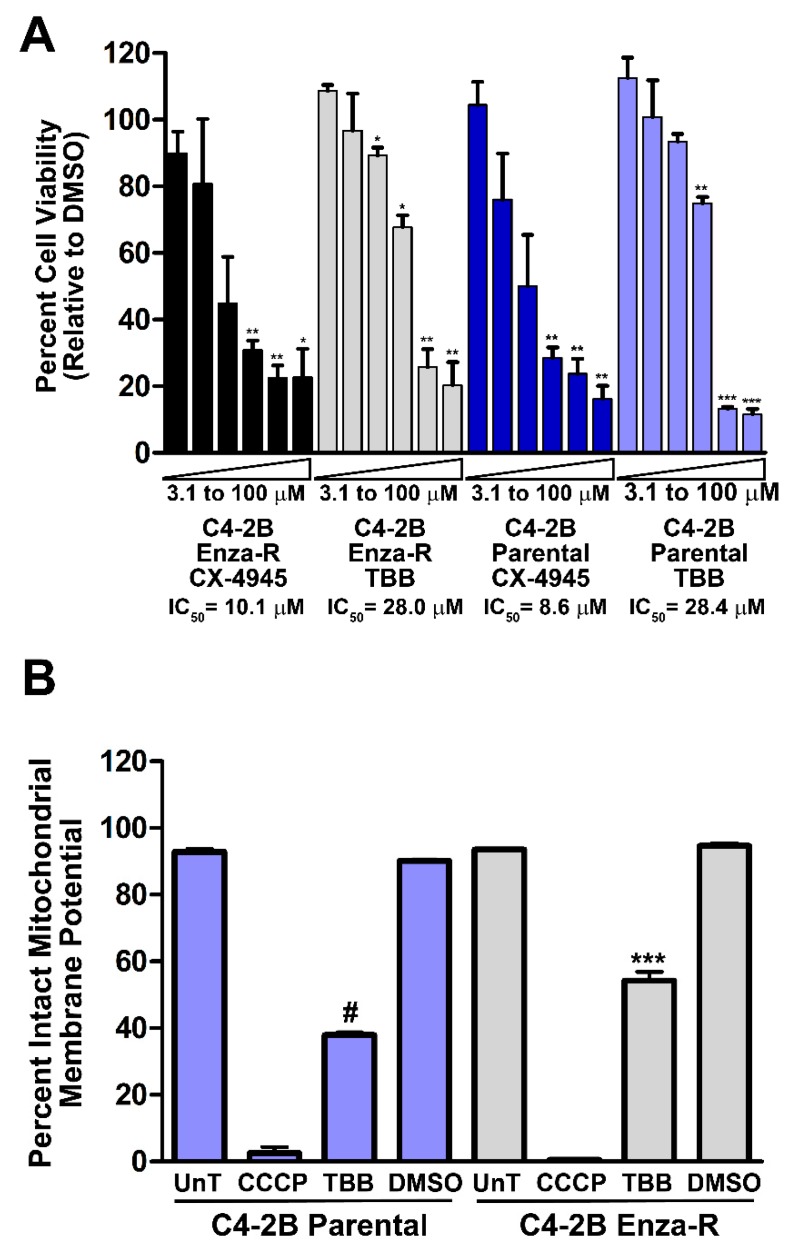
CK2 inhibition decreases cell viability and induces loss of mitochondrial membrane potential in enzalutamide-resistant C4-2B prostate cancer cells. (**A**) C4-2B parental and enzalutamide-resistant cells were treated with 2-fold dilution series of TBB, CX-4945 or equivalent concentrations of DMSO for 72 h. Cell viability was measured by MTS-based Aqueous One assay. Experiment was performed three times. Error bars indicate standard error. * p < 0.05; ** p < 0.01; *** p < 0.001; # p < 0.0001. (**B**) C4-2B parental and enzalutamide-resistant cells were treated with 50 µM TBB, or equivalent volume DMSO for 2 h. JC-1 was added to the cells 1 h after TBB or DMSO addition. CCCP treatment at 50 µM for the last 30 min of incubation was used as positive control. Experiment was performed three times. Error bars indicate standard error. *** p < 0.001; # p < 0.0001.

Prostate cancer C4-2B cells represent the lethal CRPC phenotype. Among the final modes of therapy is treatment with enzalutamide, which blocks the activity of AR in CRPC cells; however, drug resistance emerges with ensuing loss of therapeutic response. We tested the effect of inhibiting CK2 activity in enzalutamide-resistant C4-2B cells to determine their response. The results in Figure 8A show that both the parental and enzalutamide-resistant C4-2B cells treated with varying concentrations of TBB and CX-4945 demonstrated similar reduction in cell viability, suggesting that CK2 mediated cell death is not affected by the AR-targeted drug response status of these cells. Further, as shown in Figure 8B, an analogous loss of the mitochondrial membrane potential in the two types of cells in response to TBB was observed at 2 h of treatment, suggesting again that the parental and enzalutamide-resistant C4-2B cells are similarly responsive to CK2 inhibition. 

## 3. Discussion

We initiated this work to determine if elevated CK2 levels promote increased levels of AR and NFκB p65 proteins in prostate cells in general as well as PCa cells of different phenotypes. Interaction of CK2 and androgen signaling in prostate cell growth has been known for a long time [4,7]. Likewise, analysis of CK2 protein levels in human prostate tissues has been previously documented to have direct relevance to PCa disease presence and status [9,41,51], leading to proposals from our laboratory and others that CK2 is a potential target for cancer therapy [26,52,53]. Support for the importance of elevated CK2 in PCa continues to emerge from patient data. Of note, the observation that a robust co-expression of *CSNK2A1* and *AR* mRNA levels in PCa patient samples representing localized disease using TCGA data is reported here. A phosphoproteomics study using metastatic CRPC specimens found CK2 to be among the top seven enriched kinase activities in metastatic CRPC by kinase substrate enrichment analysis (KSEA) [54]. In a subsequent study, comparison of patient mRNA levels in metastatic CRPC to mRNA levels from localized PCa using TCGA data identified *CSNK2A1* (CK2α) as an inferred activated kinase [55]. Our cell-based data demonstrate that CK2 overexpression, both acute and chronic, induced elevation of total AR and NFκB p65 levels in non-transformed prostate cells. In malignant prostate cells that are androgen responsive (LNCaP) and castration-resistant (C4-2B), CK2 overexpression resulted in increased total AR protein in conjunction with higher phospho-S529 NFκB p65. These data accord with the CK2-related patient data we described here. Further, the cyclin D1 data we presented in this work suggests that the impact of CK2 was not simply one involving increased proliferation in cells with higher CK2 levels, as we have previously asserted [8,9]. Thus, elevated CK2 expression in PCa represents a potential driving factor in maintaining expression of key transcriptional factors AR and NFκB p65. 

Our results demonstrate that both parallel and divergent pathways regulating AR protein levels are activated following chemical inhibition of CK2 or its molecular down-regulation. Both modes of blocking CK2 function caused AR protein loss with reduced AR protein stability. Disparately, inhibition of CK2 activity by small molecules caused a notable loss of AR mRNA, whereas siRNA-mediated CK2 down-regulation only modestly reduced AR mRNA levels in LNCaP cells and increased AR transcripts in C4-2 cells. Regardless of its impact on AR transcript abundance, CK2 clearly plays a role in maintaining AR protein levels in prostate cells, in part through AR protein stability. Involvement of CK2 in AR function has been observed in other studies that suggested that CK2 inhibition causes altered nuclear translocation of AR and modulates AR-dependent transcription [21,23]. In contrast to our results, a previous publication noted AR-V7 protein loss in CRPC cells after CX-4945 treatment, but not loss of full length AR [24]; the basis of this difference is not clear at present. Of further note is the observation that the green tea polyphenol epigallocatechin-3-gallate (EGCG) inhibits androgen action at multiple levels resulting in inhibition of PCa growth [56]. We had previously demonstrated that EGCG inhibits CK2 in prostate cancer cells, which may represent an additional mechanism of EGCG activity on AR function [57]. 

We show an increase in various forms of ER stress signals when CK2 is inhibited; the increased BiP, CHOP and IRE1α proteins suggest marked ER stress under these conditions, and increased SQSTM1 p62 suggests involvement of autophagy in response to CK2 inhibition by TBB. The data also suggested that response may vary with the inhibitor of CK2 employed as some of the proteins showed better response with TBB than CX-4945, possibly reflecting differences in their in vivo interaction with CK2. In previous work, inhibition or molecular down-regulation of CK2 was shown to induce apoptosis in PCa cells regardless of their androgen-sensitive and -insensitive status [25]. However, another study showed that LNCaP and PC-3 cells behaved differently in response to CK2 inhibition, and suggested that the basis of this difference was that there was a crucial induction of CHOP that was responsible for causing apoptosis in LNCaP [58]. It should be noted that in our present study, we observed induction of CHOP in androgen unresponsive PCa cells (C4-2 and 22Rv1), in addition to induction of other stress signals including BiP, IRE1α, and SQSTM1 p62. In general, our results accord with observations in other systems on the ER stress and unfolded protein response to inhibition of CK2 activity [48,50]. Our observation that inhibition of CK2 results in reduced AR protein half-life and induction of ER stress is similar to a report of observed AR protein degradation in conjunction with ER stress response signals after treatment with the anti-glutamatergic drug Riluzole in different PCa cells [59]. Additionally, suppression of fatty acid synthesis was recently shown to inhibit AR full length and V7 expression with induction of ER stress in metastatic CRPC [60]. 

Crosstalk between AR and classical NFκB pathway signaling in PCa has been described in many publications, and NFκB is known to activate transcription of the AR gene [61,62]. Increased classical pathway NFκB expression raised levels of full length AR and AR-V7, and inhibition of NFκB signaling decreased AR expression [63]. We show here that both siRNA and small molecular inhibitor methods of targeting CK2 first caused loss of phospho-S529 NFκB p65, and subsequently often reduced total NFκB p65 protein. We also showed here that targeting CK2 expression through the RNAi pathway caused reduced AR and NFκB p65 levels in 22Rv1 xenograft tumors. In previous publications, we demonstrated that loss of CK2 abundance or activity in PCa xenograft tumors caused loss of total NFκB p65 overall as well as reduced phospho-S529 modification and presence of nuclear NFκB p65 [42,44,64]. It is possible that decreased AR mRNA and protein result in part from loss of NFκB-activated AR transcription in the nucleus. However, in the case of siRNA-mediated CK2 down-regulation, we detected increased AR mRNA in C4-2 cells, rendering a transcription-based mechanism unlikely to globally account for down-regulation of AR protein abundance when CK2 signaling is blocked. We propose that AR protein stability is an important pathway by which elevated CK2 promotes PCa cell survival. The data also support a possible direct relationship between CK2 activation of NFκB p65 at S529 and increased AR protein expression in malignant prostate cells. 

**Figure 9:**
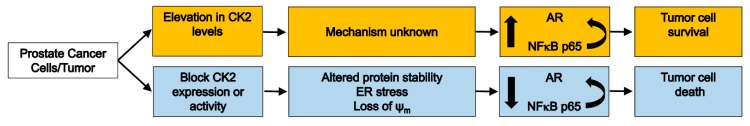
Cartoon summary of the influence of CK2 expression and activity on AR and NFκB p65 protein expression and prostate tumor cell survival as described in this work.

In summary, we have presented new data on the function of protein kinase CK2 in PCa survival. These results document new information on the effects of elevated CK2 levels in prostate cells that caused an increase in AR and NFκB p65 protein levels and enhanced activation of NFκB p65 in diverse PCa cell types. Further, we demonstrated pathways leading to cell death after blocking CK2 function in PCa cells of different phenotypes—these include an overarching impact on AR protein stability. The data are broadly summarized in Figure 9. The present work elucidates the specific link of CK2 to the pathogenesis of PCa in association with AR and NFκB function. The observation that inhibition of CK2 can exert a growth inhibitory effect on therapy-resistant PCa cells is a key point. We previously demonstrated a death program in cultured PCa cells following CK2 inhibition in which a very early event was loss of mitochondrial membrane potential at 2 h, followed by Bid and caspase cleavage events as well as cytochrome *c* release from mitochondria [65]. Here, we observed that CK2 inhibition in enzalutamide-resistant cells dramatically reduced mitochondrial membrane potential, as was similarly seen in the parental C4-2B cells. These results emphasize the possible utility of CK2 inhibition in patients who are on enzalutamide treatment for advanced cancer.

## 4. Materials and Methods

### 4.1. Cell Lines and Culture 

PC3-LN4 and C4-2 cells were obtained as described [66,67]. LNCaP and 22Rv1 cells were obtained from ATCC (Manassas, VA, USA). C4-2B parental and enzalutamide-resistant cells were a kind gift from Dr. Allen Gao (University of California at Davis, Davis, CA, USA) [68]. PC3-LN4 cells were authenticated by Johns Hopkins University genetics core facility using a 9-marker STR profile (Baltimore, MD, USA). PC3-LN4, LNCaP, C4-2, C4-2B (parental) and 22Rv1 cells were grown in RPMI-1640 with 25 mM HEPES and L-glutamine (SH30255.01; HyClone Laboratories, Logan, UT, USA) with 10% fetal bovine serum and 1% Pen/Strep; the C4-2B enzalutamide-resistant cells were additionally cultured with 20 µM enzalutamide. RWPE-1 cells were obtained from ATCC and cultured in Keratinocyte Serum Free Medium with bovine pituitary extract and epidermal growth factor (17005042; Thermo Fisher Scientific, Waltham, MA, USA). All cell lines were grown in monolayer culture in an incubator at 37 °C with 5% CO_2_. All cells had undetectable levels of mycoplasma when thawed, and were maintained in culture for up to 2 months, with the exception of the RWPE-1 stably transfected cell lines which were kept in culture for up to 84 days. For androgen-deprivation growth conditions, cells were grown in phenol-free RPMI 1640 (Quality Biological, Gaithersburg, MD, USA; 112-040-101) with 10% charcoal dextran-treated fetal bovine serum (Thermo Fisher Scientific, Waltham, MA, USA; SH30068.03).

### 4.2. siRNA and Plasmid Transfections and Stable Cell Line Production

Standard chemistry siRNAs were obtained from Dharmacon (Lafayette, CO, USA). The siCK2 guide strand sequence is 5′-auacaacccaaacuccacauuu-3′ [67]. The control siRNA (siControl) used was siNon-targeting #2 (Dharmacon; D-001810-02). Transfections of siRNA were performed using Dharmafect 2 reagent (Thermo Fisher Scientific) as described [69]. 

Transfections of plasmid DNA were performed using Lipofectamine 3000 (Thermo Fisher Scientific); for a 60 mm plate of cells, 7.5 µL of Lipofectamine 300, 10 µL of P3000, and from 4 to 6 µg of plasmid DNA were combined in a total volume of 0.5 mL per manufacturer instructions. For transient expression studies, 4 µg of FLAG-CK2α or empty donor plasmid were used, and cell pellets were collected, flash frozen on dry ice, and stored at −80 °C until processed for immunoblot analysis as described above.

The FLAG-CK2α construct for CRISPR-mediated insertion into the AAVS1 genomic site was constructed using AAVS1-SA-puro-EF1-MCS donor plasmid (System Biosciences, Mountain View, CA, USA; GE622A-1). The full length open reading frame for CK2α (NM_177559.2; Origene, Rockville, MD, USA; SC107027) was amplified by standard PCR according to recommendation of the manufacturer (Roche Diagnostics, Mannheim, Germany; 11 732 641 001) using a 5′ primer that inserted the BsrGI site and the FLAG tag in frame at the amino-terminal end (5′-gcgcctactctagatgtacagccctcgccatggactacaagg-3′) and a 3′ primer that inserted the MluI site at the 3′ end (aataaccgcggaatacgcgtttactgctgagcgccagcgg). The targeting vector used was AAVS1-gRNA/Cas9 SMartNuclease (System Biosciences; CAS601A-1). Cloning into the donor vector was performed according to manufacturer instructions using the Cold Fusion cloning kit (System Biosciences; MC010A-1). Positive clones were sequenced fully on both strands. For RWPE-1 FLAG-CK2α and empty vector stable cell line construction, 4 µg of FLAG-CK2α or empty donor plasmid combined with 2 µg of Cas9/gRNA plasmid were transfected into cells at 70% confluence. The next day, cells were split 1:5 into 5 each 60 mm plates. Six days later, each 60 mm dish was split into a 10 cm dish. On day 8 after transfection, puromycin selection began at 1 µg/mL (Gibco Laboratories, Gaithersburg, MD, USA; A11138-03) and continued for 7 days. Pooled cells were frozen back in 10% DMSO with 40% FBS after an additional 3 days of puromycin selection. Matching cell pellets (FLAG-CK2α and either parental or empty donor cells) were collected at various time points and stored at −80 °C until processed for immunoblot analysis.

### 4.3. Cell Viability Assays

CellTiter 96^®^ Aqueous One Assay (Promega Corp., Fitchburg, WI, USA) was used to assess cell viability following treatments. Cells were plated in 96-well plates (4000 cells/well for C4-2B parental; 4500 cells/well for C4-2B-EnzR) and allowed to attach overnight; enzalutamide selection was removed the days that cells were plated into 96 well plates. For TBB and CX-4945, concentrations from 100 μM to 3.1 μM were added to cells by 1:2 dilutions, and fresh drug in media was added every 24 h. DMSO was added to cells at equivalent concentrations. Aqueous One assays were performed at 24, 48, and 72 h of treatment as described [65].

### 4.4. Immunoblot Analysis

Cell pellets from cultured cells were processed in radioimmunoprecipitation assay (RIPA) buffer as previously described [45]. Tumor lysates were prepared as previously described [44]. Fifteen to 20 µg of each lysate were subjected to electrophoresis by TGX 5-15% midi gel system (BioRad, Hercules, CA, USA) or by Novex 4–12% Bis-Tris Midi gel (ThermoFisher Scientific) followed by wet tank transfer to nitrocellulose membrane, as recommended by the respective gel manufacturer. After transfer, the membranes were fully dried, rehydrated in nano-pure water, blocked for 30 min with 5% nonfat milk (Bio-Rad 170-6404) or 5% bovine serum albumin (Sigma-Aldrich Corp., Saint Louis, MO, USA, A-9647) in Tris buffered saline (TBS, pH 7.4) with 0.1% Tween 20 (TBS-T) at room temperature. Antibodies were diluted into fresh blocking buffer according to the manufacturer’s recommendations, and the membranes processed as described [45]. Antibodies used: CK2α (A300-197A) and CK2α΄ (A300-199A) from Bethyl Laboratories (Montgomery, TX, USA); CK2α΄ (CSNK2A2) from ABclonal (A1616; Woburn, MA, USA); AR (ab133273) from Abcam (Cambridge, United Kingdom); AR-V7 (AG10008) from A & G Pharmaceuticals (Columbia, MD, USA). CK2β (sc-46666), NFκB p65 P-S529 (sc-101751), AR (sc-818), and Actin (sc-1616) from Santa Cruz Biotechnology (Santa Cruz, CA, USA); NFκB p65 (6956), Cyclin D1 (12231), BiP (3177), IRE1α, CHOP (2895), SQSTM1 p62 (88588), Beta-Tubulin (2128) from Cell Signaling (Danvers, MA, USA). Proteins were detected by enhanced chemiluminescence and quantitated as described [70].

### 4.5. Quantitative Real-Time RT-PCR Analysis

Total RNA was isolated from frozen cell pellets using the RNeasy mini kit (Qiagen), including the on-column DNase digestion according to the manufacturer’s protocol, and quantitated using a NanoDrop spectrophotometer. The High-capacity cDNA Reverse Transcription Kit (Thermo Fisher Scientific) was used to synthesize cDNA from total RNA (1.5 µg) using oligo-dT primers according to the manufacturer’s protocol and with the following conditions: 10 min 25 °C; 120 min 37 °C, 5 min 85 °C. Pre-designed primers were obtained from Integrated DNA Technologies, Assay Ids: AR Hs.PT.56a.20244349; RPLP0 Hs.PT.58.20222060; B2M Hs.PT.58v.18759587. Reactions were run according to manufacturer’s specification using 96 well FAST plates on an ABI 7900HT machine. Analyses were performed using the SDS 2.3 ABI software and changes calculated according to the 2^(-∆∆Ct)^ method. RPLP0 and B2M were used as the reference gene for normalization. All results are reported as the average of reactions from two independent experiments with each sample run in duplicate. 

### 4.6. AR Protein Half Life Experiments

Cycloheximide (VWR, Radnor, PA, USA; 94271) was added at a concentration of 50 µM for all experiments. DMSO and ethanol were added to control cells at concentrations to match the addition of TBB or cycloheximide. SiRNA transfections were performed as described above, and cycloheximide was added 22 h post-transfection. Cycloheximide and TBB were added to cells simultaneously using cells at 70% confluence. Cells were collected as PBS-washed pellets, which were stored at −80 °C, at the timepoints indicated in the figure. Cell pellets were processed for immunoblot analysis as described above.

### 4.7. JC-1 FACS Analysis of Cultured Cells 

JC-1 FACS analyses were performed as previously described [65]. Three independent experiments were performed.

### 4.8. Human Prostate Cancer TCGA Data Analysis

Analysis of co-expression of CSNK2A1 and AR mRNA levels in PCa patient samples was performed using cBioPortal with a Z-score cut-off set at 1.5 and using the PanCancer Atlas data set from The Cancer Genome Atlas (n = 494 samples). 

### 4.9. Prostate Cancer Xenograft Experiment

NOD SCID gamma mice were obtained from an in-house colony. Mice were castrated under isoflurane anesthesia [45]. Seven days later, orthotopic tumors were initiated by injection of 2 million 22Rv1 cells and tumors tracked as described previously [45]. Drug preparation and tail vein injections on days 1, 4 and 7 were performed as described previously [44]. Mice were sacrificed on day 8 of the treatment schedule. The tumor take rate was 70%.

### 4.10. Statistical Analysis 

Data were summarized and analyzed using GraphPad Prism 5. Cell viabilities (ratio of CK2i to DMSO) were compared to 1 using one sample, two-sided t-tests for three independent experiments. Means and 95% confidence intervals were presented for immunoblot data. Means and standard deviations were presented for q-RT-PCR and FACS data. JC-1 FACS data for TBB compared to DMSO was analyzed by unpaired two-tailed t-test with Welch’s correction. AR protein half-lives were calculated by regression using Excel. Correlation coefficients and significance calculations for PCa patient TCGA mRNA data presented in Figure 2 were performed by cBioPortal. 

## Figures and Tables

**Table 1:** Fold-change in protein level due to increased expression of ck2α.

Cell Line	Protein Detected						
	CK2α	CK2α′	CK2β	AR	NFκB p65	NFκB p65 P-S529	Cyclin D1
**RWPE ^1^** **(stable)**	1.5 (1.1, 1.9)	1.2 (1.0, 1.3)	1.6 (0.4, 2.8)	1.5 (0.9, 2.0)	1.8 (0.9, 2.8)	0.9 (0.6, 1.2)	0.6 (0.5, 0.7)
**RWPE ^2^** **(transient)**	2.9 (2.2, 3.5)	1.9 (1.7, 2.2)	1.6 (1.3, 1.9)	1.4 (1.2, 1.9)	1.3 (0.9, 1.6)	1.2 (0.6, 1.7)	1.0 (0.9, 1.1)
**C4-2B ^2^** **(transient)**	4.4 (4.1, 4.7)	1.2 (1.0, 1.4)	1.4 (0.9, 1.8)	1.2 (0.8, 1.7)	1.0 (0.8, 1.2)	3.3 (0.2, 6.4)	1.1 (0.9, 1.3)
**LNCAP ^3^** **(transient)**	3.6 (2.5, 4.6)	1.7 (1.6, 1.7)	1.2 (1.2, 1.2)	1.4 (1.3, 1.5)	1.0 (0.8, 1.1)	1.9 (1.7, 2.2)	ND

Means and 95% confidence intervals are presented. ^1^ Numbers represent mean expression in Flag-CK2α cells relative to control empty vector cells or parental cells at three time points. ^2^ Numbers represent mean expression in Flag-CK2α cells relative to control empty vector cells (n = 3). ^3^ Numbers represent mean expression in Flag-CK2α cells relative to control empty vector cells (n = 2). ND = not determined.

**Table 2:** Fold-change in protein level due to siRNA-mediated CK2 down-regulation.

Cell Line	Time Point (h)	Protein Detected			
		CK2α	CK2α′	CK2β	AR
**LNCaP**	48	0.60 (0.48,0.72)	0.68 (0.59,0.77)	0.67 (0.59,0.76)	0.79 (0.63,0.96)
	72	0.54 (0.36,0.73)	0.43 (0.19,0.68)	0.49 (0.35,0.62)	0.70 (0.53,0.87)
**C4-2**	48	0.56 (0.47,0.65)	0.20 (0.10,0.30)	0.58 (0.53,0.63)	0.27 (0.18,0.36)
	72	0.21 (0.12,0.30)	0.10 (0.08,0.12)	0.50 (0.35,0.65)	0.15 (0.13,017)

Means and 95% confidence intervals are presented. Numbers represent mean expression in siCK2 transfected cells relative to siControl cells for two independent experiments.

**Table 3:** Fold-change in protein level of AR after CK2 inhibition.

Cell Line	Time Point (h)	TBB	CX-4945
**LNCaP**	8	0.71 (0.42, 1.00)	1.13 (0.68, 1.58)
	16	0.31 (0.11, 0.51)	0.96 (0.84, 1.08)
	24	0.36 (−0.25, 0.97)	0.62 (0.06, 1.18)
	48	0.17 (−0.06, 0.40)	0.56 (0.10, 1.01)
**C4-2**	8	0.87 (0.74, 1.00)	0.97 (0.73, 1.21)
	16	0.69 (0.44, 0.94)	0.89 (0.88, 0.90)
	24	0.62 (0.46, 0.78)	0.67 (0.30, 1.04)
	30	0.33 (−0.19, 0.85)	1.92 (0.16, 3.68)
**22Rv1**	8	0.84 (0.81, 0.87)	0.70 (0.49, 0.91)
	16	0.79 (0.73, 0.85)	0.75 (0.58, 0.92)
	24	0.77 (0.76, 0.78)	0.75 (0.48, 1.02)
	30	0.65 (0.62, 0.68)	0.74 (0.68, 0.80)

Means and 95% confidence intervals are presented. Values represent mean expression in TBB- or CX-4945-treated cells relative to DMSO control cells for two independent experiments.

**Table 4:** AR mRNA expression levels after CK2 inhibition or down-regulation.

Cell Line	Time Point (h)	TBB	siCK2
**22Rv1**	8	0.86 ± 0.07	ND
	16	0.75 ± 0.06	ND
	24	0.60 ± 0.10	ND
	48	0.29 ± 0.23	ND
**C4-2**	8	0.71 ± 0.04	ND
	16	0.36 ± 0.01	ND
	24	0.35 ± 0.03	ND
	48	0.48 ± 0.02	1.25 ± 0.09
	72	ND	1.80 ± 0.19
**LNCaP**	48	0.12 ± 0.02	ND
	72	ND	0.82 ± 0.04

mRNA expression normalized to geometric mean of RPLP0 and B2M and calculated by 2^(-∆∆Ct)^ method. Expression values are relative to DMSO for TBB treatment and relative to siControl for siCK2 values. Standard deviation represents two independent experimental samples. ND = not determined.
